# Targeting progressive multiple sclerosis: Toward mechanism‐informed precision medicine

**DOI:** 10.1111/joim.70125

**Published:** 2026-07-21

**Authors:** Fredrik Piehl, Gonçalo Castelo‐Branco, Maja Jagodic, Tomas Olsson

**Affiliations:** ^1^ Department of Clinical Neuroscience Karolinska Institutet Centre for Molecular Medicine Karolinska University Hospital Stockholm Sweden; ^2^ Centre for Neurology Academic Specialist Centre Stockholm Sweden; ^3^ Department of Neurology Karolinska University Hospital Stockholm Sweden; ^4^ Division of Molecular Neurobiology Department of Medical Biochemistry and Biophysics Karolinska Institutet Stockholm Sweden

**Keywords:** biomarkers, disease progression, disease‐modifying therapy, lifestyle factors, multiple sclerosis

## Abstract

Multiple sclerosis has undergone a therapeutic revolution over the past three decades. Randomized clinical trials and real‐world data demonstrate that modern disease‐modifying therapies substantially reduce relapse rates and acute inflammatory activity detected by magnetic resonance imaging (MRI). However, disability accumulation increasingly occurs independent of relapse activity, highlighting progression biology as the principal unmet need. Converging epidemiological and molecular evidence supports a pivotal role for Epstein–Barr virus (EBV) infection in disease initiation, whereas later stages appear dominated by brain‐intrinsic mechanisms, including compartmentalized inflammation, microglial activation, failure of remyelination and accelerated biological ageing. Population‐based cohorts demonstrate that early high‐efficacy therapy improves long‐term outcomes, yet the risk of progression rises markedly after midlife despite effective relapse suppression. Emerging biomarkers, such as serum neurofilament light chain, glial fibrillary acidic protein, paramagnetic rim lesions and advanced quantitative MRI metrics, now enable more granular monitoring of progressive pathology. Integration of imaging, fluid biomarkers, genetics and machine learning offers opportunities for individualized benefit–risk stratification. Brain‐penetrant Bruton's tyrosine kinase inhibitors, CD40 ligand‐targeting biologics, refined B‐cell‐depleting strategies and emerging chimeric antigen receptor T‐cell therapies represent promising approaches to target different aspects of compartmentalized inflammation and smoldering disease biology. Future management will require mechanism‐informed treatment algorithms that align therapeutic choice with dominant disease drivers while incorporating comorbidity management, de‐escalation strategies and potential EBV‐targeted preventive approaches to optimize outcomes across the entire disease course.

## Graphical abstract

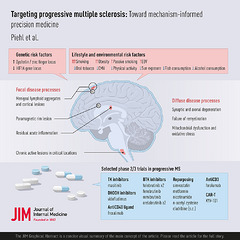

## Introduction

Multiple sclerosis (MS) is a chronic inflammatory and neurodegenerative disease of the central nervous system (CNS) and remains a leading cause of neurological disability in young adults [1, 2]. Although disease‐modifying therapies (DMTs) have transformed the management of inflammatory disease activity [3, 4], worsening of disability still occurs in a substantial proportion of people with MS [5]. Increasing evidence suggests that MS is best conceptualized as a dynamic disease spectrum in which inflammation driven by adaptive immune activity originating in the periphery gradually gives way to age‐dependent, localized and diffuse inflammatory and neurodegenerative processes [6]. This evolving view of MS biology underscores the need to better characterize mechanisms driving progression.

Traditionally, MS has been classified according to clinical course into relapsing–remitting MS (RRMS), secondary progressive MS (SPMS) following an initial relapsing phase, and primary progressive MS (PPMS) in the absence of relapses [1, 2]. Radiologically isolated syndrome and clinically isolated syndrome represent early disease stages, preceding clinical onset and marking the first clinical presentation, respectively. MS may also be stratified by age at onset into pediatric‐onset MS (<18 years), adult‐onset MS, and late‐onset MS (>50 years) [7]. However, these classifications incompletely capture the underlying biological heterogeneity, underscoring the added value of mechanism‐based classifications.

Disease susceptibility reflects complex gene–environment interactions, with important contributions from HLA risk variants, smoking, vitamin D deficiency, obesity, and Epstein–Barr virus (EBV) infection [8]. Notably, accumulating longitudinal and molecular epidemiological data indicate that EBV infection is a near‐universal antecedent of MS and thus likely represents a prerequisite for disease initiation in otherwise susceptible individuals [9, 10, 11]. However, much less is known about factors influencing the disease course, and only recently have genetic and lifestyle determinants associated with disease severity started to be mapped.

Indeed, the MS disease course is highly variable between individuals, but evidence suggests a spectrum rather than a set of discrete subtypes, with substantial biological and clinical overlap across phenotypes. Although earlier disease stages and younger age are associated with higher relapse frequency, the age at reaching key disability milestones is similar across phenotypes, indicating that age is a dominant driver of long‐term disability accumulation [6, 7, 12, 13, 14]. Nevertheless, with increasing opportunities for intervention, this variability underscores the need for improved biomarkers and integrative frameworks to define progression and guide personalized treatment strategies.

The scope of this review is to highlight the evolving understanding of the biological divide underpinning inflammatory and progressive MS, and to synthesize advances in imaging and fluid biomarkers, genetic and lifestyle risk factors, and emerging therapeutic strategies related to this distinction. Emphasis is placed on the highly variable and often fluid transition from inflammation‐driven disease to progression dominated by neurodegeneration and compartmentalized pathology, as well as on how integration of multimodal data may enable mechanism‐informed, precision medicine approaches to improve long‐term outcomes.

## Controlling inflammation

Over the past three decades, the landscape of MS treatment has changed considerably with the introduction of increasingly effective DMTs [4]. More than 20 therapies have demonstrated significant reductions in relapse rates and magnetic resonance imaging (MRI) lesion activity in randomized clinical trials (RCTs) in RRMS, whereas only two DMTs (Ocrelizumab and Siponimod) have been shown to reduce the risk of disability worsening in PPMS and SPMS, respectively [3]. Secular trends further show declining relapse rates in both trials and real‐world RRMS cohorts [15]. Notably, high‐efficacy therapies with high treatment persistence, particularly anti‐CD20 B cell–depleting therapies, have created a clinical scenario in which the vast majority of patients remain relapse‐free during the first decade after treatment initiation [16].

Despite these advances, safety considerations remain central to the benefit–risk balance of MS therapies. Early initiation of high‐efficacy treatment may improve long‐term outcomes but requires careful evaluation of therapy‐related risks [16, 17]. For example, alemtuzumab, a monoclonal antibody targeting CD52‐expressing lymphocytes, is now rarely used due to rare but serious complications, including autoimmune and cardiovascular events, whereas daclizumab, targeting the interleukin‐2 receptor, was withdrawn altogether due to severe inflammatory adverse events, including encephalitis and meningoencephalitis. Overall, with existing highly efficacious therapies, increased susceptibility to infections represents the major safety concern [18]. Natalizumab, which blocks lymphocyte trafficking into the CNS, increases the risk of opportunistic infections such as progressive multifocal leukoencephalopathy and herpes zoster, whereas anti‐CD20 therapies are primarily associated with bacterial infections [19, 20]. Importantly, treatment‐related risks tend to increase with age, highlighting the need for careful monitoring, comorbidity management, and de‐escalation strategies in aging populations [18, 21]. Family planning considerations and therapeutic sequencing, including rebound disease after discontinuation of lymphocyte migration modulators (natalizumab and sphingosine‐1‐phosphate receptor modulators), underscore that risk management extends beyond drug‐specific adverse effects to treatment transitions and patient‐specific factors [22, 23, 24].

## Confronting progression

Although increasingly effective immunomodulatory DMTs have been introduced, sustained disability worsening still occurs in approximately 10%–20% of patients within the first decade after treatment initiation, and differences between DMTs in reducing this risk are substantially smaller than their effects on relapse reduction [16, 25]. Age at treatment initiation appears to be an important modifier, as subgroup analyses of RCTs show that the relative benefit of high‐efficacy therapy is primarily observed in individuals younger than 40 years [26]. This is consistent with the notion that, with increasing age and disease duration, progressive biology becomes more prominent [7, 27]. Compared with earlier relapsing stages, the progressive phase is characterized by compartmentalized inflammation behind a relatively intact blood–brain barrier, astrocyte and microglial activation, chronic active lesions, synaptic degeneration, mitochondrial dysfunction, oxidative stress, and impaired remyelination [6, 28, 29, 30, 31, 32].

**Fig. 1:**
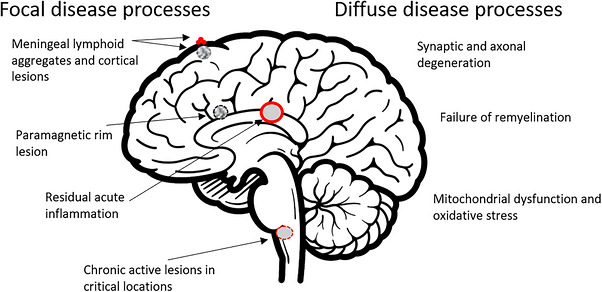
Localized and diffuse pathological drivers of MS progression. Schematic illustration of the interplay between localized and diffuse pathological processes underlying progressive worsening in MS. Focal chronic active lesions situated in anatomically critical locations, such as along the corticospinal tract (red dashed circle), can disproportionately drive clinical worsening, particularly motor disability. Slowly expanding or paramagnetic rim lesions (black dashed line) can also be found throughout the brain. Cortical lesions (grey circle), often adjacent to aggregates of lymphoid tissue in the meninges (red dots), are another feature observed in longstanding MS. In parallel, residual acute inflammation (solid red circle), although less intense than in earlier disease stages, may still contribute to gradual worsening. Finally, widespread synaptic and neuroaxonal degeneration, microglial activation, and mitochondrial dysfunction contribute to progressive tissue loss across the brain and spinal cord. Both localized and diffuse components are influenced by intrinsic MS‐related inflammatory and neurodegenerative mechanisms, as well as by extrinsic factors associated with aging and comorbidities. Together, these converging processes shape the heterogeneous trajectory of disease progression.

Reflecting this evolving understanding of MS biology, new clinical concepts have emerged to describe disability accumulation beyond relapses. Progression independent of relapse activity (PIRA) refers to disability worsening occurring in the absence of a preceding relapse and has become an important outcome measure in contemporary clinical trials and observational studies [5, 33, 34]. However, PIRA is primarily defined using the Expanded Disability Status Scale (EDSS) and may not fully capture the multidimensional deterioration of physical and cognitive function observed in treated MS populations. The EDSS is an ordinal scale ranging from 0 (normal neurological examination) to 10 (death due to MS), with a strong emphasis on ambulation and limited sensitivity to upper limb function, cognition, and other non‐motor domains. To better align clinical terminology with underlying disease mechanisms, the concept of smoldering MS has been proposed [35]. Smoldering MS reflects a convergence of pathological processes, including chronic active lesions, diffuse gray and white matter damage, meningeal inflammation, synaptic loss, and neuroaxonal degeneration (Fig. 1). Clinically, these processes may manifest as fatigue, slowed cognitive processing speed, reduced neurological reserve, and gradual functional decline [36]. However, there is currently no universally accepted clinical or biological definition of smoldering MS, which limits its use as a standardized or definitive outcome in clinical trials and routine practice.

## Beyond EDSS: Expanding outcome assessment in progressive MS

The EDSS remains the most widely used outcome measure in RCTs, observational studies, and regulatory settings [37]. However, EDSS lacks sensitivity to subtle, relapse‐independent worsening, particularly early in the disease course. Its strong emphasis on ambulation limits the detection of upper limb dysfunction, cognitive impairment, fatigue, and other patient‐relevant domains [35]. In addition, floor and ceiling effects and inter‐ and intra‐rater variability reduce its responsiveness to incremental change [1].

To address these limitations, composite measures, such as the MS Functional Composite and EDSS‐Plus, have been developed [38, 39]. These incorporate the EDSS (only EDSS‐Plus), the 9‐Hole Peg Test (a measure of upper‐limb function), the Timed 25‐Foot Walk and the Symbol Digit Modalities Test (only the MS Functional Composite), which assesses cognitive processing speed. Although these measures are more sensitive, and in some respects more dynamic, their implementation in routine clinical practice remains limited.

Patient‐reported outcomes and digital monitoring may offer complementary perspectives. Instruments, such as the Fatigue Scale for Motor and Cognitive Functions and the MS Impact Scale‐29, capture fatigue and quality‐of‐life aspects important to patients [40, 41]. However, relationships between traditional physician‐administered and patient‐reported scales are complex. In large population‐based cohorts, cross‐sectional analyses show that different metrics capture overlapping but distinct dimensions of disease burden, even though their trajectories often remain relatively stable over time [25, 42, 43]. Emerging data from wearable devices and smartphone sensors provide yet another dimension in a multidimensional assessment framework [44]. In MS, continuous monitoring of step count, gait and upper‐limb function correlates with clinician‐rated and patient‐reported functioning and may detect subtle decline not captured by EDSS [45, 46]. Large prospective platforms, such as Floodlight Open (>1,000 participants), demonstrate feasibility and clinically relevant associations using smartphone‐based assessments [47]. As this is a rapidly evolving field, validation across large longitudinal cohorts will be required to establish robustness, standardization, and clinical utility before broad clinical implementation, a process that will inevitably take time.

## Imaging and soluble biomarkers of progressive disease biology

### Traditional imaging metrics

MRI has played a central role in both MS care and research, not only in diagnosis but also in enabling therapeutic development and monitoring treatment response. The field has evolved rapidly in recent years, with major advances in hardware, sequence design, and image processing that allow the extraction of increasingly detailed biological information. According to the 2021 European and North American MS imaging network (MAGNIMS–CMSC–NAIMS) consensus recommendations, MRI is essential for monitoring disease activity and treatment effectiveness beyond the diagnostic phase [48]. Standardized MRI protocols, with 3D FLAIR as a core sequence, are critical for reliable longitudinal comparison (Table 1). New or enlarging T2 lesions remain the most practical markers of inflammatory activity, as routine follow‐up can often be performed without contrast if a recent reference scan is available.

**Table 1:** MRI sequences for monitoring multiple sclerosis.

Category	MRI sequence	What it shows/Captures	Clinical use/Best for	References
Clinical routine/Core clinical	FLAIR	T2‐like but with suppressed CSF signal	Core sequence for diagnosis and monitoring; highest sensitivity for MS lesions, particularly periventricular and juxtacortical	[48]
Clinical routine/Core clinical	T1‐weighted (pre‐contrast)	Hypointense “black holes” reflecting chronic tissue damage and axonal loss	Assessing tissue integrity, chronic lesion burden, and brain atrophy	[48]
Clinical routine/Core clinical	T1‐weighted + gadolinium	Blood–brain barrier breakdown	Detection of active lesions; used selectively	[48]
Clinical routine/Core clinical	T2‐weighted imaging	Increased water content (edema, inflammation, demyelination); CSF appears bright	Detection of total lesion burden (complementary to FLAIR)	[48]
Clinical routine/Adjunct	Spinal cord MRI (T2, STIR, PD)	Focal or diffuse cord lesions; inflammatory demyelination	Diagnostic, prognosis, and selected monitoring (esp. progressive disease)	[48]
Clinical routine/Adjunct	DWI	Restricted diffusion in acute lesions (non‐specific)	Differential diagnosis (e.g., stroke, abscess)	[48]
Research/Advanced MRI	Volumetric T1 (atrophy)	Whole brain/grey matter atrophy	Neurodegeneration and disability prediction; research and clinical trials	[49]
Research/Advanced MRI	SWI/QSM	Magnetic susceptibility effects (iron deposition, paramagnetic rims, venous structures)	Detecting chronic active lesions (PRLs); marker of progressive disease	[49]
Research/Advanced MRI	Cortical imaging (DIR, PSIR)	Improved grey–white contrast enabling cortical lesion detection	Detection of cortical lesions; marker of progressive disease	[49]
Research/Advanced MRI	Myelin imaging (SyMRI, REMyDI, MTR)	Myelin content quantification; remyelination failure	Diffuse damage; progressive MS; remyelination trials	[49, 50, 51, 52]
Research/Advanced MRI	DTI/Neurite density imaging	Microstructural axonal integrity; white matter tract damage	Early axonal injury; research and clinical trials	[49]
Research/Advanced MRI	Perfusion imaging	Cerebral blood flow alterations	Altered perfusion and metabolic dysfunction	[49]
Advanced/Experimental	7 T MRI	Higher resolution for cortical lesions, PRLs, central vein sign	Advanced phenotyping (research setting)	[49]
Advanced/Experimental	AI‐based analysis	Multimodal integration of structural changes	Research (e.g., BrainAge) and risk stratification	[53]

Abbreviations: 7T, 7 Tesla; AI, artificial intelligence; CSF, cerebrospinal fluid; DIR, double inversion recovery; DTI, diffusion tensor imaging; DWI, diffusion‐weighted imaging; FLAIR, fluid‐attenuated inversion recovery; MRI, magnetic resonance imaging; MS, multiple sclerosis; MTR, magnetization transfer ratio; PD, proton density; PRLs, paramagnetic rim lesions; PSIR, phase‐sensitive inversion recovery; QSM, quantitative susceptibility mapping; REMyDI, rapid estimation of myelin for diagnostic imaging; STIR, short tau inversion recovery; SWI, susceptibility‐weighted imaging; SyMRI, synthetic magnetic resonance imaging.

However, conventional inflammatory markers are less informative for reflecting processes underlying progressive disease biology. Brain atrophy measured with standardized 3D T1‐weighted imaging is the most established imaging correlate of neurodegeneration, with regional measures, such as thalamic and cortical gray matter atrophy or upper cervical spinal cord cross‐sectional area, appearing more sensitive than global total brain parenchymal fraction [48, 54]. It has also been observed that individuals with progressing motor deficits often have a preexisting lesion in a critical location along the corticospinal tract [55]. Notably, the enlargement of the choroid plexus has also been shown to correlate with progressive disease and risk of disability worsening [56, 57]. However, all volumetric measures are vulnerable to scanner changes and typically require prolonged follow‐up before meaningful interpretation at the individual level is possible.

### Emerging imaging metrics

**Fig. 2:**
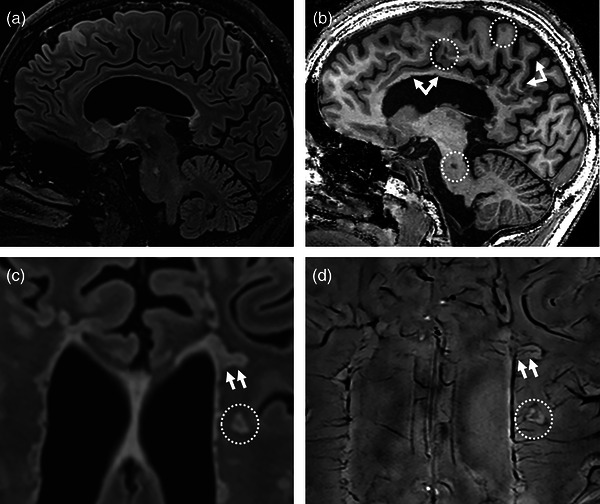
Ultra–high‐field MRI features of progressive multiple sclerosis. 7 T MRI of a 34‐year‐old individual with MS (symptom onset at age 26, diagnosis at age 27) who developed a progressive disease course at age 32, despite initiation of highly effective monoclonal antibody therapy at diagnosis and subsequent hematopoietic stem cell transplantation at age 28. Notably, no new focal lesions appeared after treatment initiation. FLAIR (a) and T1‐weighted (b) images demonstrate several features characteristic of progressive MS, including numerous cortical lesions and lesions in anatomically critical locations (white dashed circles in b), as well as diffuse thinning of the corpus callosum and cerebral cortex (white arrows in b). Higher‐magnification views of periventricular white matter (c, FLAIR; d, susceptibility‐weighted imaging) reveal lesions with paramagnetic rims (dashed circles in c and d), many of which are centered on a central vein (white arrows in c and d).

The limitation with traditional MRI measures of progression has driven efforts to identify cross‐sectional markers of progressive pathology. Paramagnetic rim lesions and slowly expanding lesions show particular promise, as they correspond to neuropathologically defined chronic active lesions characterized by an outer rim of activated myeloid cells, ongoing axonal injury, and limited lymphocytic infiltration (Fig. 2) [58, 59]. Recent single‐cell and spatial transcriptomics studies are starting to elucidate the molecular and cellular composition of such expanding lesions [60, 61, 62, 63, 64]. Moreover, recent spatial transcriptomic and positron emission tomography imaging studies have further identified “broad rim lesions,” marked by extensive innate immune activation and linked to rapid disease progression [65]. Although validated standardized acquisition and semi‐automated analysis protocols still need to be developed for routine clinical implementation, quantitative susceptibility mapping represents a promising approach [66]. Some studies further suggest that cerebral hypoperfusion may represent a non‐inflammatory mechanism contributing to neuroaxonal injury and long‐term disability in MS, highlighting perfusion imaging as a potential tool for understanding disease progression [67].

Emerging high‐field 7‐T MRI represents yet another important expansion of the frontier, as greater magnetic field strength improves the sensitivity for detection of cortical lesions and smaller expanding lesions and the central vein therein (Fig. 2) [68]. Moreover, quantitative microstructural imaging metrics, including myelin and neurite integrity [50, 51, 69], have shown promise for capturing diffuse pathology that would otherwise go undetected using conventional imaging methods. Quantitative sequence techniques, such as synthetic MRI, enable the estimation of myelin integrity and may capture diffuse tissue damage beyond focal lesions (Fig. 2) [49, 50, 51, 52]. At a systems level, lesion network mapping and disconnectome analyses are emerging as approaches to link lesion location to functional network disruption [70], potentially clarifying mechanisms underlying symptoms such as fatigue.

Finally, artificial intelligence offers a meta‐analytic framework. Brain age modeling, derived from machine learning applied to MRI data, provides a condensed marker of overall structural brain integrity [71]. Recent larger and longitudinal studies demonstrate robust correlations between brain age and clinical disability [53], supporting its potential as an imaging biomarker for progression and personalized medicine. Examples and a summary of relevant MRI techniques and outcomes are given in Fig. 2 and Table 1.

### Soluble biomarkers of progressive disease biology

Compared with imaging, soluble biomarkers have emerged more recently as an important complementary modality for monitoring both inflammatory and progressive MS aspects [72]. Markers of intrathecal immunoglobulin synthesis, such as immunoglobulin M and G oligoclonal bands and the κ‐free light chain index, reflect B‐cell–driven adaptive immune activity and are valuable not only diagnostically but also prognostically, particularly in early relapsing disease [73, 74, 75]. Cerebrospinal fluid (CSF) CXCL13, a B cell chemokine, captures active B‐cell recruitment and correlates with inflammatory lesion activity and relapse risk, supporting its role as a marker of inflammatory disease activity [76]. However, broader clinical implementation requires reliable blood‐based assays, and few biomarkers show a strong correlation between CSF and peripheral blood compartments [77, 78].

### Neurofilament light chain

Neurofilament light chain (NfL), measurable in both CSF and serum, is a sensitive indicator of neuroaxonal injury. It reflects acute inflammatory activity as well as subclinical tissue damage and predicts short‐term relapse risk and MRI activity [79]. Associations with long‐term brain atrophy and disability progression are more modest and appear phenotype‐dependent [80, 81]. However, in both mixed MS cohorts and RRMS‐only populations, NfL elevations several years before the event predict risk of disability worsening better than levels measured immediately preceding clinical worsening [82].

### Glial fibrillary acidic protein

Glial fibrillary acidic protein (GFAP) captures astrocytic activation and astrogliosis, processes increasingly recognized as central to the biology of progressive MS and is the principal intermediate filament of mature astrocytes. It is upregulated during reactive astrogliosis and can be reliably quantified in blood. Elevated serum GFAP levels independently predict an increased risk of PIRA, while serum sNfL primarily associates with inflammatory activity and relapse risk [83]. Thus, GFAP and NfL provide complementary information, capturing distinct but overlapping aspects of MS disease biology.

To improve interpretability in clinical practice, *z*‐score transformation references have been developed for both NfL and GFAP [84]. In both cases, variability in the reference population increases with advancing age, which negatively affects their informative value [83, 85]. It is important to be aware that these markers are not specific to MS, and potential confounding effects of comorbidities must be considered in their clinical interpretation (Table 1) [79, 86].

### Additional soluble protein markers

Markers of microglial activation, including CHI3L1, CHIT1, and TREM2, are highly relevant as they reflect innate immune activation associated with smoldering pathology and chronic active lesions [72, 87]. Indeed, higher CSF levels of CHIT1 have been associated with faster disability progression [88]. Moreover, in a larger panel of CSF proteins reflecting myeloid activation, CHIT1 emerged as the most informative marker for predicting future disability accumulation [89]. SERPINA3—elevated in the CSF of people with MS, particularly in progressive patients [90]—is another emerging biomarker, as it represents a hallmark of disease‐associated glia, especially within oligodendrocyte lineage cells [91, 92]. Notably, a recent study identified blood levels of N‐formylated peptides—endogenous agonists of formyl peptide receptor 1—as being associated with MS disease progression and representing a potential therapeutic target [93]. Technologies such as proximity extension assays combined with next‐generation sequencing (e.g., Olink), as well as large‐scale mass spectrometry–based proteomics, are rapidly expanding the repertoire of CSF and plasma biomarkers in MS, linking them to specific disease stages and underlying neuropathological processes, and enabling differentiation from other neurological diseases [78, 94]. However, based on the historically slow adoption of existing biomarkers into clinical practice, it is likely that the translation of these advances into routine MS care will take time.

### Non‐protein biomarkers

More recently, non‐protein markers have begun to be explored as biomarkers of MS disease biology. Lipid metabolites, including oxysterols and ceramides, may reflect altered myelin turnover and neuronal injury [95]. Circulating microRNAs and DNA methylation patterns have also been investigated as indicators of immune activation, neurodegenerative pathways, and accelerated biological ageing. Analysis of cell‐free chromatin modifications is emerging as a diagnostic and potentially prognostic tool in cancer [96, 97], with potential applicability to neurological diseases. In addition, metabolomic studies have identified alterations in pathways such as tryptophan–kynurenine and energy metabolism that may be linked to progressive disease mechanisms [98]. However, these approaches remain largely exploratory and require further validation.

## Risk factors for MS progression; genes and lifestyle

Defining risk factors for disability progression is important, as it can improve our understanding of the underlying biological mechanisms, potentially paving the way for new therapeutic approaches and informing public health strategies targeting modifiable lifestyle factors. However, in contrast to disease risk, which is typically considered a binary outcome, functional disability develops along a spectrum, varying in both severity and the functional domains affected. This complexity makes its determinants more difficult to decipher, particularly as comorbidities may further complicate the progression and manifestation of disability.

### Gene‐environment impact on MS risk

Research on risk factors has provided extensive insight into genetic susceptibility to MS. Genome‐wide association studies have identified more than 200 non‐HLA loci, most with modest effect sizes [99]. The strongest associations lie within the HLA complex, where HLA‐DRB115:01 confers approximately a threefold increased risk, whereas HLA‐A02 is protective [99, 100]. In addition to genetic factors, environmental and lifestyle exposures—including EBV infection, smoking, organic solvents, obesity, low sun exposure, and head trauma contribute to disease risk, with some interacting with HLA alleles to further increase susceptibility [8]. Risk loci are predominantly expressed in immune cells, supporting roles in adaptive and innate immunity. However, emerging epigenomic data indicate that CNS‐resident cells, particularly oligodendroglia, may also exhibit open chromatin at select risk loci, thereby potentially influencing processes such as proliferation, differentiation, and immune cell recruitment [101].

### Genetics of MS severity

As none of the known MS risk loci has been clearly linked to disease progression [102], the mechanisms underlying disability worsening likely differ from those driving susceptibility and may instead involve CNS‐intrinsic processes. Consistent with this, the genetic contribution to progression appears substantially smaller, accounting for approximately 13%–17% of the variance [102]. Although with the caveat that large, well‐characterized cohorts still remain limited, these findings suggest that lifestyle and environmental factors may play a greater role in progression than in disease susceptibility.

Evidence supporting CNS‐intrinsic mechanisms comes from a large genome‐wide association study by the International MS Genetics Consortium, including >12,000 pwMS and >9000 controls [102]. The study identified a locus encompassing *DYSF* (dysferlin) and a zinc finger protein gene, with expression largely restricted to the CNS, associated with a ∼3.7‐year delay in the need for a walking aid and greater neurocognitive reserve. More recently, a candidate‐gene study examining iron metabolism and oxidative stress pathways identified a protective *HIF1A* allele linked to more favorable 20‐year disability outcomes [103].

### Environmental and lifestyle factors associated with severity

EBV has been proposed as a potential driver also of chronic disease mechanisms, although direct evidence linking EBV to disability progression remains limited [10, 11, 104]. EBV persists lifelong in B cells and may therefore reside within CNS‐compartmentalized B cells, potentially escaping depletion by monoclonal therapies with limited CNS penetration. Molecular mimicry between EBV antigens (e.g., EBNA1) and CNS targets, such as ANO2, has been described, with sustained antibody and T‐cell responses detectable years after diagnosis [105, 106, 107]. In contrast, serological evidence of infection with human cytomegalovirus has been associated with reduced MS risk and certain aspects of disease progression [108, 109].

In contrast to MS susceptibility, relatively few studies have examined lifestyle and environmental factors influencing disability progression (Table 2). Such studies rarely establish causation and may be confounded by reverse causation but remain important for individuals seeking guidance on modifiable potential risk factors. Among modifiable factors, smoking is the most consistently associated with worse outcomes, including faster disability progression, whereas cessation is linked to more favorable trajectories [110]. Passive smoking and continued smoking after diagnosis worsen outcomes, whereas use of oral tobacco has been associated with more favorable disability trajectories, suggesting lung irritation rather than nicotine as a key mechanism [111]. Other inhaled irritants, such as air pollution and organic solvents, may exert similar effects [112, 113].

**Table 2:** Genetic, lifestyle, and environmental factors associated with MS progression.

(A) Genetic factors associated with MS progression			
Factor	Level of evidence	Direction of association and selected outcomes	References
Dysferlin/zinc finger locus (rs10191329)	+++	Risk allele vs. non‐risk allele: increased risk of disability progression (24‐week confirmed disability worsening, HR 1.10, 95% CI 1.02–1.18) and faster time to disability milestones (EDSS 6, HR 1.22, 95% CI 1.09–1.38), with evidence of increased brainstem and cortical pathology	[102]
*HIF1A* gene locus (rs11621525)	+++	Protective variant vs. non‐carriers: increased likelihood of favorable disease course (defined as EDSS <4; OR 1.75, 95% CI 1.39–2.27 in Italian cohort; OR 1.27, 95% CI 1.05–1.49 in Swedish cohort), with reduced smouldering pathology and neuroaxonal injury	[103]

(B) Lifestyle and environmental factors associated with MS progression			
Smoking	+++	Ever‐smokers vs. never‐smokers: increased risk of reaching disability milestones (EDSS 4, HR 1.34, 95% CI 1.12–1.60); current smokers vs. never‐smokers (HR 1.64, 95% CI 1.33–2.02); ex‐smokers vs. current smokers: reduced risk (HR 0.65, 95% CI 0.50–0.83)	[110, 111, 114]
Passive smoking	+	Current passive smoking vs. never exposed increased risk of reaching disability milestones (EDSS 3, HR 1.49, 95% CI 1.29–1.74); past exposure vs. never exposed: no significant association (HR 1.03, 95% CI 0.93–1.12)	[111]
Oral tobacco	+	Current use vs. never use: reduced risk of reaching disability milestones (EDSS 3, HR 0.85, 95% CI 0.72–0.99); past use vs. never use: no significant association (HR 0.80, 95% CI 0.63–1.00)	[111]
EBV	+	Increased EBV titers (study‐specific cut‐offs): associated with higher relapse rate (rate ratio 1.26, 95% CI 1.03–1.56) and linked to reaching EDSS 3 in univariable analyses; associations with EDSS 4 and EDSS 6 were not consistently significant	[109]
CMV	+	Increased CMV titers (study‐specific cut‐offs): associated with reduced risk of reaching disability milestones (EDSS 4, HR 0.95, 95% CI 0.91–0.99) and lower risk of conversion to SPMS (HR 0.94, 95% CI 0.90–0.99)	[109]
Obesity	++	Obesity (>30 kg/m^2^) vs. healthy BMI (18.5–24.9): increased risk of reaching disability milestones (EDSS 3, HR 1.43, 95% CI 1.17–1.75; EDSS 4, HR 1.40, 95% CI 1.07–1.73), along with worse patient‐reported outcomes and increased MRI activity	[115, 116]
Physical activity	+	Higher physical activity at diagnosis vs. low activity: reduced risk of confirmed disability worsening (HR 0.77, 95% CI 0.61–0.97 for moderate; HR 0.71, 95% CI 0.54–0.94 for moderate‐high; HR 0.64, 95% CI 0.46–0.90 for high activity)	[117]
Sun exposure	+	Low vs. high sun exposure at diagnosis: increased risk of reaching disability milestones (EDSS 3, HR 1.35, 95% CI 1.02–1.79)	[118]
Fish consumption	+	Higher intake vs. lower intake: reduced risk of reaching disability milestones (EDSS 3, HR 0.55, 95% CI 0.39–0.79)	[119]
Alcohol consumption	+	Low–moderate vs. no consumption: reduced risk of reaching disability milestones (EDSS 3, HR ∼0.78–0.83); high consumption: no significant association	[120]

*Note*: The number of “+” symbols indicates the level of evidence: +++ denotes robustly replicated findings; ++ indicates replicated evidence; and + reflects evidence from single studies or strong suspicion based on circumstantial data.

Abbreviations: CI, confidence interval; CMV, cytomegalovirus; EBV, Epstein–Barr virus; EDSS, Expanded Disability Status Scale; HIF1A, hypoxia‐inducible factor 1‐alpha; HR, hazard ratio; MS, multiple sclerosis; OR, odds ratio; SPMS, secondary progressive multiple sclerosis.

Obesity during the disease course has been associated with faster progression, increased disability risk, and greater cognitive decline, with synergistic effects observed with smoking [115, 121]. Limited sun exposure has also been linked to worsening disability, although reverse causation is a concern [118]. Dietary factors may also play a role, as higher fish consumption at diagnosis has been associated with reduced progression risk [119].

Regarding protective factors, higher physical activity at diagnosis and sustained over time is associated with reduced disability progression, whereas pre‐diagnosis activity shows no clear effect [117]. Low‐to‐moderate alcohol consumption has similarly been linked to more favorable outcomes compared with abstinence, whereas high intake shows no clear association [120]. Although these observational findings should be interpreted cautiously, they provide a preliminary basis for practical lifestyle guidance in MS.

## Emerging therapies targeting progressive disease biology

As in other neurodegenerative conditions, progressive MS remains one of the greatest therapeutic challenges in neurology [122, 123]. Despite major advances in suppressing inflammatory aspects in relapsing disease, numerous Phase II and III trials in progressive MS over the past two decades have yielded neutral or only modestly positive results [3, 124, 125]. Agents targeting broad immunosuppression, neuroprotection, or putative neurodegenerative pathways have failed to demonstrate meaningful reductions in confirmed disability progression, the primary outcome measure recognized by regulatory authorities. This likely reflects both an incomplete understanding of the underlying biology and limitations of traditional trial designs and outcome measures [122, 123]. The growing evidence supporting a role for EBV in the pathogenesis of MS has stimulated interest in targeted interventions [126], although evidence of effectiveness remains preliminary [127].

### BTK inhibitors

Given the emerging recognition of the role of compartmentalized inflammation, attention has shifted toward additional therapeutic targets, including innate immune mechanisms within the CNS, particularly microglial activation and chronic active lesions. Brain‐penetrant Bruton's tyrosine kinase (BTK) inhibitors represent the drug class that has advanced furthest in clinical development. Several second‐generation BTK inhibitors are currently in Phase II or III development for relapsing and/or progressive MS, including tolebrutinib, fenebrutinib, remibrutinib, orelabrutinib, and BIIB091 (Table 3) [125, 128]. These agents inhibit BTK signaling in both B cells and myeloid lineage cells, including microglia, thereby potentially targeting adaptive as well as innate immune pathways implicated in compartmentalized CNS inflammation.

**Table 3:** Selected Phase 2/3 trials in progressive MS.

Substance	Trial identifier	Phase	MS subtype	Start year	Completion	Size	Results	References
**BTK inhibitors**								
Tolebrutinib	NCT04411641	3	nrSPMS	2020	2024	1131	Tolebrutinib vs. placebo: reduced risk of disability worsening (HR 0.69, 95% CI 0.55–0.88; 22.6% vs. 30.7%; *p* = 0.003). Safety signal for hepatotoxicity	[129]
Tolebrutinib	NCT04458051	3	PPMS	2020	2025	767	Tolebrutinib vs. placebo: no difference in risk of disability worsening (EDSS‐based; HR 0.86, 95% CI 0.64–1.15; *p* = 0.32. Safety signal for hepatotoxicity	[130]
Fenebrutinib	NCT04544449	3	PPMS	2020	2025	985	Non‐inferiority vs. ocrelizumab for risk of disability worsening (HR 0.88, 95% CI 0.75–1.03). No reported safety signal*	
Remibrutinib	NCT07225504	3	nrSPMS	2025	Est. 2030	Est. 1275		
Orelabrutinib	NCT07067463	3	PPMS	Est. 2026	Est. 2030	Est. 705		
Orelabrutinib	NCT07299019	3	SPMS	Est. 2026	Est. 2030	Est. 990		
**TK inhibitor**								
Masitinib	NCT05441488	3	PPMS/nrSPMS	2022	Est. 2028	Est. 800		
**DHODH inhibitor**								
Vidofludimus	NCT05054140	2	PMS	2021	2025	450	No significant effect on primary endpoint (brain volume change), but reduced risk of disability worsening (∼20% overall; ∼30% in PPMS subgroup). No reported safety signal*	[131]
**Repurposing**								
Simvastatin	NCT03387670	3	SPMS	2018	2024	964	Simvastatin vs. placebo: no reduction in risk of disability worsening (HR 1.13, 95% CI 0.91–1.39; 40% vs. 36%; *p* = 0.26). One case of rhabdomyolysis in active arm	[132]
Metformin	NCT05893225	2	PMS	2023	Est. 2027	Est. 120		
Nicotinamide	NCT05740722	2	PMS	2023	Est. 2027	Est. 300		
N‐acetyl cysteine	NCT05122559	2	PMS	2022	Est. 2027	Est. 98		
Cladribine (s.c.)	NCT05961644	2/3	SPMS	2022	Est. 2027	Est. 188		
**AntiCD40 ligand**								
Frexalimab	NCT06141486	3	nrSPMS	2023	Est. 2028	Est. 900		
**AntiCD3**								
Foralumab	NCT06292923	2	nrSPMS	2023	Est. 2026	54		
**CAR‐T**								
KYV‐101	NCT06384976	2	PMS	2024	Est. 2029	Est. 120		

*Note*: Asterisk (*) denotes preliminary top‐line data. Information is based on publicly available trial registry listings. Unless otherwise stated, the primary endpoint is time to confirmed disability progression (typically assessed over 12 or 24 weeks).

Abbreviations: BTK, Bruton's tyrosine kinase; CAR‐T, chimeric antigen receptor T cell; DHODH, dihydroorotate dehydrogenase; EDSS, Expanded Disability Status Scale; NCT, clinical trial identifier (ClinicalTrials.gov); nrSPMS, non‐relapsing secondary progressive multiple sclerosis; PMS, progressive multiple sclerosis (including PPMS and SPMS); PPMS, primary progressive multiple sclerosis; s.c., subcutaneous; SPMS, secondary progressive multiple sclerosis; TK, tyrosine kinase.

Despite a strong mechanistic rationale, clinical results with BTK inhibitors have been mixed. Evobrutinib failed to demonstrate superiority over teriflunomide in reducing relapse rates in RRMS Phase III trials, leading to the discontinuation of its development [133]. In contrast, tolebrutinib has shown encouraging effects on disability outcomes, particularly in progressive disease. In the HERCULES trial in non‐relapsing SPMS, tolebrutinib reduced the risk of confirmed disability worsening by 31% compared with placebo (HR 0.69; 95% CI, 0.55–0.88; *p* = 0.003) [129], representing the first positive Phase III trial in this setting. These findings indeed support the concept that CNS‐penetrant BTK inhibition can modify PIRA. Notably, the effect size compares favorably with the 25% risk reduction observed with ocrelizumab in the ORATORIO PPMS trial [134], despite an older population and lower baseline inflammatory activity in HERCULES.

The notion that BTK inhibitors may differentially influence MS disease processes is further supported by the GEMINI RRMS Phase III trials. In these studies, tolebrutinib failed to reduce annualized relapse rates or contrast‐enhancing lesions compared with teriflunomide, yet pooled post hoc analyses suggested a lower risk of confirmed disability worsening (HR 0.71; 95% CI, 0.53–0.95) [135]. This contrasts with established DMTs, where effects are typically greatest on inflammatory activity and more modest on disability progression [3]. Results in PPMS have been less consistent. Tolebrutinib did not significantly delay disability worsening in the PERSEUS trial [130], whereas fenebrutinib met primary endpoints in both the FENhance (RRMS; annualized relapse rate) and FENtrepid (PPMS; disability worsening) trials [136, 137]. These discrepancies may reflect differences in trial design or patient characteristics, although subgroup analyses suggest that features such as paramagnetic rim lesions may predict response [138], consistent with targeting chronic active inflammation. No clear increase in serious infections has been observed to date, but reports of liver injury with tolebrutinib highlight the need for careful safety monitoring [129].

### CD40 ligand–receptor modulation

Targeting the CD40–CD40 ligand (CD40L) pathway represents another promising therapeutic strategy in MS. To date, frexalimab—an anti‐CD40L monoclonal antibody—is the only such compound to have undergone Phase II evaluation [139], and it is currently being investigated in Phase III trials in RRMS and non‐relapsing SPMS. CD40L—expressed on activated T cells—binds to CD40 on B cells, dendritic cells, and myeloid cells, thereby amplifying adaptive immune responses, promoting antibody production, and enhancing antigen presentation. Importantly, the CD40 signaling cascade is also implicated in EBV‐related immune responses, further increasing its relevance as a therapeutic target in MS [140, 141], and contributes to activation of innate immune pathways, including proinflammatory cytokine production by myeloid cells [142]. This dual role at the interface of adaptive and innate immunity makes CD40L an attractive target in MS, where both arms of the immune system drive disease activity and progression. However, as a monoclonal antibody, frexalimab has limited penetration across the blood–brain barrier compared with small‐molecule BTK inhibitors and is therefore likely to primarily modulate peripheral immune activation rather than directly target compartmentalized CNS inflammation, representing a potential limitation in progressive MS.

### Refining B‐cell targeting

As previously noted, anti‐CD20 therapies have transformed MS treatment, although their impact in progressive MS remains modest compared with relapsing disease [143, 144], a notion further supported by the disappointing results of a recent high‐dose trial [145]. This discrepancy may reflect compartmentalized inflammation within the CNS, including ectopic lymphoid‐like structures that are relatively inaccessible to peripherally acting therapies. Although anti‐CD20 agents reduce circulating and CSF B cells, their ability to achieve deep and sustained depletion within CNS tissue remains limited, underscoring the need for strategies that more effectively target intrathecal inflammation. Emerging approaches include targeting broader B‐cell populations (e.g., CD19), bispecific antibodies, and enhanced CNS delivery platforms such as Brainshuttle technologies [146]. However, these strategies remain at early stages of development, with none yet advanced to Phase II trials or beyond.

### Cell‐based therapies

Experiences with hematopoietic stem cell transplantation in progressive MS have been mixed, although this intervention may have some benefit in younger individuals and when applied earlier in the disease course [147, 148, 149]. Novel cellular therapies represent a new and potentially less harmful strategy to access the CNS and eliminate tissue‐resident immune cells. Chimeric antigen receptor T‐cell (CAR‐T) therapy is an emerging approach that involves genetically engineered autologous T cells targeting specific immune cell antigens [150]. Current CAR‐T strategies in MS primarily focus on B‐cell depletion through targets such as CD19 and B‐cell maturation antigen, reflecting the central role of B cells in disease pathogenesis. Early clinical experience and ongoing phase I trials suggest that CAR‐T cells may achieve deeper and potentially more sustained immune depletion than conventional anti‐CD20 therapies, with the additional advantage of penetrating the CNS [150, 151]. However, CAR‐T therapy is complex, costly, and associated with significant risks, including serious neurological adverse events [152]. Despite these limitations, it may offer meaningful benefits in carefully selected patients with severe, treatment‐refractory MS in whom conventional therapies have failed. Case reports of KYV‐101, a fully human CD19‐directed CAR‐T therapy, have been published [153], and a Phase II study in SPMS and PPMS is planned (ClinicalTrials.gov identifier: NCT06384976).

### Reparative therapies

Neuroprotection and improved remyelination remains a relatively underexplored and as yet unsuccessful therapeutic avenue in MS. Despite strong biological rationale, efforts at targeting inhibitory pathways for oligodendrocyte precursor cell differentiation, or providing metabolic support have failed to demonstrate clinically meaningful benefit in Phase III trials. These include monoclonal antibodies such as opicinumab and elezanumab [154, 155], as well as small molecules such as high‐dose biotin and bexarotene [156, 157].

More recently, attention has also shifted toward repurposing existing drugs [158]. This can be exemplified by clemastine fumarate, a CNS‐penetrant antihistamine that has shown modest signals of improving nerve conduction in chronically demyelinated optic nerve tracts in a proof‐of‐concept trial [159]. However, its clinical utility is limited by side effects such as increased fatigue, which is particularly problematic in MS, where fatigue often is a disabling symptom. A recent setback was the failure of simvastatin in a Phase III trial in SPMS [132], despite a promising signal in an earlier study [132]. Metformin has shown a potentially beneficial mechanism of action in rejuvenating oligodendrocyte precursor cells [160] and is currently being investigated in ongoing clinical trials; however, a first smaller such trial failed to provide clear evidence of benefit [161]. Overall, whereas repurposed agents and novel compounds continue to be investigated, the lack of robust clinical efficacy to date highlights the challenges of translating remyelination strategies into meaningful therapeutic benefit in MS.

## Conclusion

Disability worsening in MS remains a major unmet therapeutic challenge despite substantial advances in controlling acute inflammatory disease activity. Clinical worsening in MS reflects a complex interplay between residual inflammatory activity, compartmentalized CNS‐driven pathology, comorbid conditions, and processes related to biological ageing, all of which variably contribute across individuals and disease stages. Although traditional DMTs have primarily targeted peripheral adaptive immunity, growing insights into progressive disease biology—including the roles of glial activation (not only microglia, but also oligodendroglia and astrocytes, that transition to disease‐associated states), chronic active lesions, impaired remyelination, and neurodegeneration—are reshaping therapeutic strategies.

Recent developments—including CNS‐penetrant drugs, cellular therapies, and attempts at remyelination—underscore both the promise and the challenges of addressing progression. At the same time, advances in imaging, fluid biomarkers, and computational risk modeling now enable more refined characterization of disease processes, creating opportunities for a more personalized approach to treatment. This may involve earlier and optimized use of high‐efficacy therapies to reduce long‐term progression risk; incorporation of lifestyle and comorbidity management; and rational treatment sequencing—such as, for example, induction with anti‐CD20 therapies followed by CNS‐penetrant BTK inhibitors.

Future progress will depend on how well therapeutic strategies can be aligned with the dominant disease mechanisms in individual patients, as well as on the design of clinical trials enriched for populations most likely to benefit from specific interventions. Together, these developments suggest that the field is entering a phase in which biologically informed, precision medicine approaches—which may include prevention, targeted immunomodulation, and neuroprotective or regenerative strategies—can optimize long‐term outcomes across the entire disease course.

## Author contributions

**Fredrik Piehl**: Conceptualization; Writing—original draft; funding acquisition; writing—review and editing. **Maja Jagodic**: Conceptualization; funding acquisition; writing—review and editing. **Tomas Olsson**: Conceptualization; funding acquisition; writing—review and editing. **Gonçalo Castelo‐Branco**: Conceptualization; funding acquisition; writing—review and editing.

## Conflict of interest statement

The authors declare no conflicts of interest.

## Data Availability

Data sharing not applicable to this article as no datasets were generated or analysed during the current study.
